# Efficacy and Safety of Aidi Injection as an Adjuvant Therapy on Advanced Breast Cancer: A Systematic Review and Meta-Analysis of Randomized Controlled Trials

**DOI:** 10.1155/2020/2871494

**Published:** 2020-08-21

**Authors:** Yihui Chai, Yunzhi Chen, Wen Li, Zhong Qin, Jie Gao, Zhibin Jiang, Yuhong Ge, Liancheng Guan, Mengzhi Zhang, Huaiquan Liu, Haiyang Yu, Qingxue Wang, Changfu Yang

**Affiliations:** ^1^Department of Preclinical Medicine, Guizhou University of Traditional Chinese Medicine, Guiyang, Guizhou, China; ^2^Center for Traditional Chinese Ethnic Minority Medicine, Guizhou University of Traditional Chinese Medicine, Guiyang, Guizhou, China; ^3^Center for Translational Medicine, Guizhou University of Traditional Chinese Medicine, Guiyang, Guizhou, China; ^4^Department of Pharmacy, Guizhou University of Traditional Chinese Medicine, Guiyang, Guizhou, China; ^5^Second Affiliated Hospital, Guizhou University of Traditional Chinese Medicine, Guiyang, Guizhou, China

## Abstract

**Background:**

Aidi injection (ADI) is being used widely for breast cancer in China. However, the efficacy and safety of it need to be summarized. We conducted a systematic review and meta-analysis to compare ADI and non-ADI treatment for advanced breast cancer.

**Methods:**

We searched PubMed, EMBASE, CNKI, SinoMed, and CENTRAL from inception to Jan 2020 for randomized controlled trials (RCTs) with diagnosis of advanced breast cancer that compared the efficacy of ADI with non-ADI treatment. Two researchers screened the literature, extracted data, and evaluated risk of bias separately. The primary outcomes were overall response rate (ORR) and disease control rate (DCR). The secondary outcomes included the QOL, immune cells, and adverse events. Review Manager software was used for estimating risks of bias of included studies, data analysis, and plotting. The sensitivity analysis and the publication bias test were performed using the *R* language. *I*^2^ and chi-square tests were used to estimate heterogeneity. If *P* > 0.1 or *I*^2^ < 40%, the fixed-effect model meta-analysis was performed. A random or fixed-effect analysis was used depending on the heterogeneity testing. Weighted mean difference (WMD) or standard mean difference (SMD) was used for analysis of continuous data, and the rate ratio (RR) was calculated for the dichotomous variable, respectively.

**Results:**

We included 14 studies with 1006 patients diagnosed as advanced breast cancer in total. The pooled effect showed that ADI increased ORR in advanced BC patients as an add-on therapy with little heterogeneity (*RR* = 1.14, 95% CI 1.03–1.27). DCR in BC patients could not be improved by ADI. ADI improved the KPS score in BC patients compared with chemotherapy alone (MD = 3.26, 95% CI 1.74–4.78). There were no improvements on immune markers except CD4/CD8 and NK%. Serum tumor markers CEA and CA153 were decreased while treated with ADI, but only one trial was involved. ADI decreased the numbers of myelosuppression in advanced BC patients, and AST, ALT, *γ*-GT, and CK-MB were all decreased. The sensitivity evaluation indicated that the result of the pooled effect size had good stability.

**Conclusion:**

This meta-analysis suggested that based on the existing evidence, treatment with ADI significantly changed the ORR of patients with advanced BC and improved their quality of life with few side effects. However, more randomized trials involving larger samples should be considered, and detailed mechanisms are needed to be uncovered.

## 1. Introduction

Breast cancer (BC) is one of the most usual malignant tumors among women worldwide which results in high rates of morbidity and mortality [1, 2]. Over the past two decades, the incidence rate of BC has been increasing constantly [3, 4]. Accumulating evidence showed that genes, proteins, and several pathways are involved in the occurrence and progression of BC, and the precise molecular mechanisms are still unclear. To date, surgery is the first choice for early-stage BC patients, but most clinically diagnosed advanced BC patients are forced to accept chemotherapy [5], radiotherapy [6], endocrine therapy [7], or biotherapy [8]. The management of the disease is primarily to improve quality of life (QOL) and prevent disease from recurring.

Although targeted add-on therapy with monoclonal antibodies such as trastuzumab or pertuzumab has been proved to be efficacious in specific types of BC, the high costs still slowed down the widespread use in developing countries of the world. Therefore, effective and affordable adjunct therapies are needed. Aidi injection (ADI) is a compound preparation injection of Chinese herbs (Z52020236, CFDA), which is composed of the extracts from *Panax ginseng* C. A. Mey, *Astragalus propinquus* Schischkin, *Acanthopanax senticosus* (Rupr. Maxim.) Harms, and *Mylabris phalerata* Pallas [9]. According to a study on chemical constituents in the Aidi injection, 22 chemical components were detected and isolated [10]. These compounds are astragaloside, ginsenoside, eleutheroside, coniferin, etc. Previous studies showed that ADI could significantly improve the clinical response and QOL in patients with non-small cell lung cancer (NSCLC) [11] and gastric cancer. Several clinical trials also revealed that ADI could reduce the toxicity of chemotherapy in breast cancer [12, 13]. However, the efficacy on BC has been inconclusive due to a lack of summary. Therefore, we performed a systematic review and meta-analysis. Findings from such a study may help determine whether to use ADI as an add-on therapy on BC.

## 2. Methods

### 2.1. Protocol and Registration

The protocol of the present review was registered in the International Platform of Registered Systematic Review and Meta-Analysis Protocols (Inplasy, https://inplasy.com/) and was reported in accordance with PRISMA [14] (Preferred Reporting Items for Systematic Reviews and Meta-Analyses). The registration number is INPLASY202040170, and the DOI number is 10.37766/inplasy2020.4.0170.

### 2.2. Search Strategy

We conducted an online search for trials from inception up to Jan 2020, in PubMed (https://www.ncbi.nlm.nih.gov/pubmed), EMBASE (http://www.embase.com), CNKI (http://www.cnki.net/), SinoMed (http://www.sinomed.ac.cn/), and the Cochrane Central Register of Controlled Trials (CENTRAL) (http://onlinelibrary.wiley.com/cochranelibrary/) with the search terms “(Breast Neoplasms [MH] OR breast neoplasm^*∗*^[TIAB] OR breast carcinoma^*∗*^[TIAB] OR breast tumor^*∗*^[TIAB] OR breast tumor^*∗*^[TIAB] OR breast cancer^*∗*^[TIAB]) AND (Aidi injection[TIAB]),” following the demonstration of Cochrane handbook (ZM and LH). In addition, we performed handsearches of the references of all identified articles and relevant reviews (GJ).

### 2.3. Study Selection

Eligible clinical trials were defined based on the following criteria: (1) randomized controlled trials of advanced breast cancer (parallel groups or cross-over design); (2) age >18 years; (3) intervention with Aidi injection as an add-on therapy compared with conventional chemotherapy; (4) reported ORR and adverse events or at least one additional outcome.

Exclusion criteria: (1) animal or cell research; (2) observational studies; (3) reviews, letter to the editor, or case reports; (4) duplicates.

Two authors, respectively, reviewed the titles and abstracts (LW and CY). If there were discrepancies between the present reviewers, another author (QZ) was consulted to reach a consensus as the third investigator.

### 2.4. Data Collection Process

We extracted data from each selected study, including the name of the first author, publication year, geographical location, study design, cases, participants, doses, outcomes, and statistical methods. We followed the recommendations for reporting by the Preferred Reporting Items for Systematic Reviews and Meta-Analyses guidelines [14] (PRISMA). The quality of individual records was assessed according to the Cochrane handbook.

### 2.5. Outcomes

The primary endpoint was the overall response rate [15] (complete remission + partial remission, ORR) and the disease control rate (complete remission + partial remission + stable disease, DCR). Secondary outcomes included the QOL, immune cells, and adverse events.

### 2.6. Statistical Analysis

Review Manager software (version 5.3; Cochrane Collaboration, Oxford, UK) was used for estimating risks of bias of included studies, data analysis, and plotting. The sensitivity analysis and the publication bias test were performed using the *R* language. *I*^2^ and chi-square tests were used to estimate heterogeneity. If *P* > 0.1 or *I*^2^ < 40%, the fixed-effect model meta-analysis was performed. When there was a high degree of heterogeneity, a random-effect analysis was used. For each group, the Aidi injection group was compared to placebo or other active chemotherapy. Weighted mean difference (WMD) or standard mean difference (SMD) was used for analysis of continuous data, and the rate ratio (RR) was calculated for the dichotomous variable, respectively.

## 3. Results

### 3.1. Study Description and Risk of Bias

**Figure 1:**
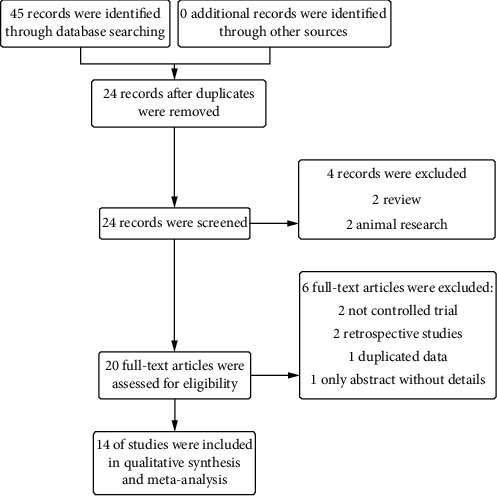
Search and selection of clinical trials assessing the efficacy and safety of ADI on advanced BC.

**Figure 2:**
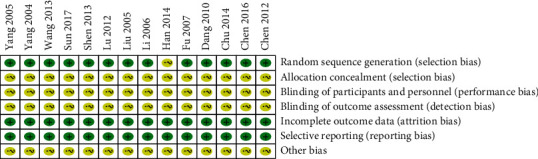
Risk of bias of included studies.

By using the search strategy mentioned above, a total of 24 trials were identified after duplicated records were removed. After screening the title and the abstracts, we retrieved the full texts of 20 records, of which 14 were ultimately included in our analysis involving 1006 participants totally. The details of the exclusions are shown in Figure 1. In total, 14 trials were included in the present study, and the characteristics of the trials are shown in Table 1. Most of the included trials showed relatively low to medium quality. The Cochrane handbook [28] was used to evaluate the risk of bias for RCTs (Figure 2). The treatments of the 14 included articles were ADI plus chemotherapy.

### 3.2. Primary Outcomes

**Figure 3:**
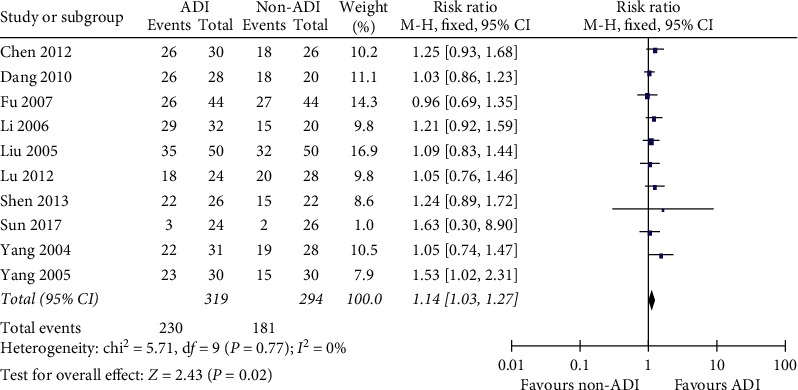
ADI increased ORR in advanced BC patients as an add-on therapy (*RR* = 1.14, 95% CI 1.03–1.27; chi^2^ = 5.71, *P*=0.77; *I*^2^ = 0%).

**Figure 4:**
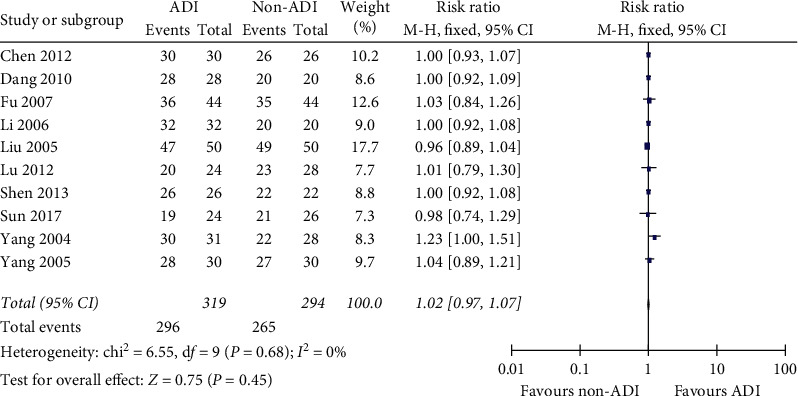
ADI did not improve DCR in advanced BC patients as an add-on therapy (*RR* = 1.02, 95% CI 0.97–1.07; chi^2^ = 6.55, *P*=0.68; *I*^2^ = 0%).

Ten trials reported ORR and DCR as the main outcome. The pooled effect showed that ADI increased ORR in BC patients as an add-on therapy with little heterogeneity (*RR* = 1.14, 95% CI 1.03–1.27; chi^2^ = 5.71, *P*=0.77; *I*^2^ = 0%; Figure 3). DCR in BC patients could not be improved by ADI as an add-on therapy (*RR* = 1.02, 95% CI 0.97–1.07; chi^2^  = 6.55, *P*=0.6; *I*^2^ = 0%; Figure 4).

### 3.3. Secondary Outcomes

**Figure 5:**
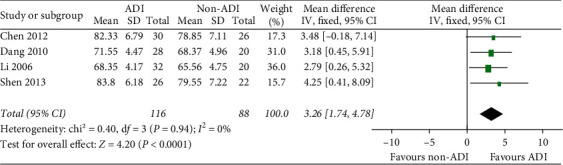
ADI improved the KPS score in advanced BC patients as an add-on therapy (MD = 3.26, 95% CI 1.74–4.78; chi^2^ = 0.4, *P*=0.94; *I*^2^ = 0%).

ADI plus chemotherapy improved the KPS score in BC patients compared with chemotherapy alone (MD = 3.26, 95% CI 1.74–4.78; chi^2^ = 0.4, *P*=0.94; *I*^2^ = 0%; Figure 5). There were no improvements on CD3%, CD4%, and CD8%. The CD4/CD8 ratio was higher while treated with ADI with a high heterogeneity (MD = 0.32, 95% CI 0.07–0.58; chi^2^ = 48.88, *P* ≤ 0.01; *I*^2^ = 88%). NK% data showed the same trend with CD4/CD8. Serum tumor markers CEA and CA153 were decreased while treated with ADI, but only one trial was involved (Table 2).

### 3.4. Adverse Events

**Figure 6:**
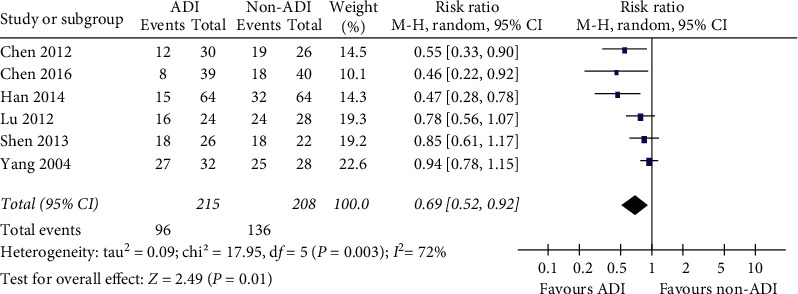
ADI decreased the numbers of myelosuppression in BC patients as an add-on therapy (*RR* = 0.69; 95% CI 0.52–0.92; *I*^2^ = 72%; *P*=0.003).

ADI decreased the numbers of myelosuppression in advanced BC patients as an add-on therapy (*RR* = 0.69, 95% CI 0.52–0.92; chi^2^  = 17.95, *P*=0.003; *I*^2^ = 72%; Figure 6). AST, ALT, *γ*-GT, and CK-MB were all decreased by ADI treatment. No other side effects were recorded during the studies. Details are shown in Table 3.

### 3.5. Publication Bias

**Figure 7:**
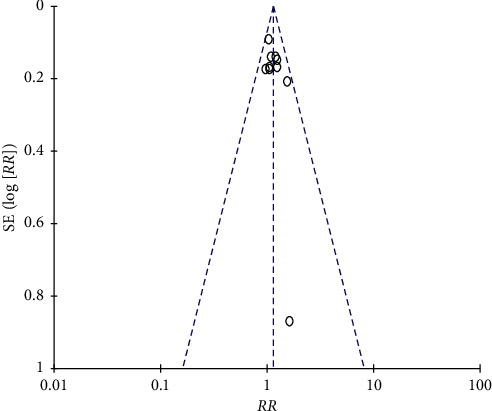
Publication bias. There was no publication bias in the included studies.

No obvious publication bias was found through the funnel plot (ORR) (Figure 7). Egger's test showed the result of the linear regression test of the funnel plot asymmetry: *t* = 1.4319, *df* = 8, and *P* value = 0.1901. The result indicated that there was no publication bias.

### 3.6. Sensitivity Analysis

**Figure 8:**
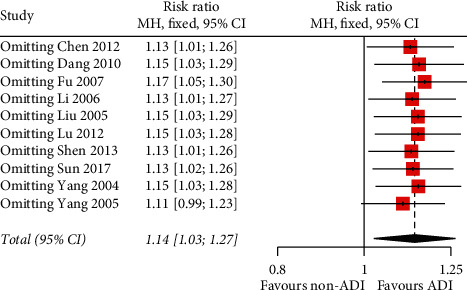
Sensitivity analysis showing that the result had good stability.

The sensitivity was evaluated through excluding the poor and overestimated studies about the main outcome ORR. The analysis indicated that the result of the pooled effect size had good stability (Figure 8).

## 4. Discussion

BC is commonly discovered among women worldwide of which the incidence rate has been increasing constantly. Advanced breast cancer patients do not have many choices but to accept chemotherapy. During a long-term clinical practice, traditional Chinese medicines have played important roles in treating some types of tumors. However, the molecular mechanisms are poorly discovered. ADI is a compound injection of Chinese herbs which is widely used in treating malignant tumors including breast cancer.

The present meta-analysis suggested that based on the existed evidence, treatment with ADI significantly changed the ORR of patients with advanced BC but did not obviously increase the DCR. There were also improvements on quality of life, and an increase in the KPS score was observed. To some extent, the immune system was improved because the CD4/CD8 ratio and NK cells were higher while treated with ADI. However, CD3, CD4, and CD8 did not change because of one study [26] which induced high heterogeneity and showed totally reversed effect to other studies. The participant's age, intervention, and duration were not significantly different from others'. ADI seemed to be safe for patients. ADI decreased the numbers of myelosuppression, AST, ALT, *γ*-GT, and CK-MB in BC patients as an add-on therapy. No obvious publication bias was found through the funnel plot (ORR).

The mechanism of ADI on BC was suggested that ADI significantly inhibited the proliferation of MCF-7 cells in a dose-dependent manner [29] and the miRNA might serve as potentially therapeutic targets. The modulation of miRNA expression is an important mechanism of ADI inhibiting breast cancer cell growth. Another experiment reported that ADI could inhibit proliferation, promote apoptosis and necrosis of tumor cells, and significantly reduce the cell diameter [30]. But, the research is limited, so more studies should better be involved in and discover the underlying mechanisms.

Several limitations of this meta-analysis should be mentioned. First, the quality of included trials was relatively low, some of which did not report the details of blinding and allocation concealment. This might induce bias of the results. Little research studies provided survival data. Previous studies showed good effect of ADI on patients with BC which may be a potential drug as an adjunct therapy. However, additional high-quality RCTs and larger sample sizes may lead to more reliable results. Second, the records of survival terms were seldomly reported, and we were not able to calculate the overall survival of specific year. Furthermore, the therapeutic duration and designs were not identical, which may lead to heterogeneity. Subgroup analysis was not performed because included articles were limited and difficult to be grouped.

## 5. Conclusion

In summary, this meta-analysis suggested that based on the existing evidence, treatment with ADI significantly changed the ORR of patients with advanced BC and improved their quality of life with few side effects. More randomized trials involving larger samples should be considered, and detailed mechanisms are needed to be uncovered.

## Figures and Tables

**Table 1:** Characteristics of included trials.

Trials	Design	No. of cases T/C	Age T/C	KPS T/C	TNM	Treatment	Control	Outcomes
Yumeng [16]	RCT	24/26	55.08 ± 10.32/54.12 ± 10.75	86.25 ± 5.76/87.50 ± 5.52	I–IV	Aidi 100 ml/d/1–7 q 21d + CEF or CAF	CEF or CAF	SAS, SDS, QLQC30, ORR, AEs
Weiming [13]	RCT	39/40	46.73 ± 14.2/45.98 ± 15.78	>50#	III-IV	Aidi 100 ml/d/1–8 q 21d + CEF	CEF	ICs, QoL, BMs, AEs
Yonghong [12]	RCT	64/64	46.7 ± 20.3	#	I–IV	Aidi 100 ml/d/1–8 q 21d + CEF	CEF	ICs, AEs
Liwang et al. [17]	RCT	78/62	52.5 (24∼76)/51.2 (20∼70)^*∗*^	#	I–IV	Aidi 100 ml/d/1–14 + CEF or CAF	CEF or CAF	ICs
Mei and Li [18]	RCT	23/23	52 (36∼64)^*∗*^	#	I–III	Aidi 100 ml/d/1–7 q 21d + CEF	CEF	VEGF
Sandi et al. [19]	RCT	26/22	42.27 ± 6.32/42.23 ± 6.7	84.23 ± 5.78/84.55 ± 5.96	IIA–IIIC	Aidi 60 ml/d/1–4 q 14d + TC-P	TC-P	ORR, QoL, AEs
Chuanhui et al. [20]	RCT	24/28	57.21 ± 3.52/55.66 ± 3.43	#	IIB–IIIB	Aidi 80 ml/d/1–15 q 21d + TAC	TAC	ORR, ICs
Zhuorong et al. [21]	RCT	30/26	42.47 ± 7.85/42.54 ± 8.10	83.67 ± 6.15/84.62 ± 5.82	II-III	Aidi 60 ml/d/1–4 q 14d + AC-T	AC-T	ORR, QoL, AEs
Xiangguo and Lin [22]	RCT	28/20	36.2 ± 3.6/37.5 ± 4.2	72.87 ± 4.69/71.89 ± 5.03	I–IIIA	Aidi 100 ml/d/1–10 q 21d + CTF	CTF	ORR, QoL, AEs
Ling and Xiaoge [23]	RCT	44/44	42 (32∼63)/48 (31∼65)	#	I–IV	Aidi 100 ml/d/1–15 q 28d + NP	NP	ORR, DCR, TTP, AEs
Xiangqiand Shaobo [24]	RCT	32/20	46.2 ± 2.6/44.5 ± 3.2	70.78 ± 4. 40/71.19 ± 4.53	I–IIIA	Aidi 100 ml/d/1–10 q 21d + CEF	CEF	ORR, QoL, AEs
Wenjuan [25]	RCT	30/30	48.4/47.6	#	III-IV	Aidi 100 ml/d/1–10 q 21d + CAF	CAF	ORR, ICs, QoL
Zhenzhen [26]	RCT	50/50	45^*∗*^	#	II-III	Aidi 100 ml/d/1–14 q 21d + CAF	CAF	ORR, ICs, QoL, AEs
Ling [27]	RCT	31/28	54.2 (32∼69)/53.5 (31∼70)	#	II–IV	Aidi 50 ml/d/1–15 q 21d + NT	NT	ORR, ICs, QoL, AEs

BC: breast cancer; T: treatment; C: control; ORR: overall response rate; DCR: disease control rate; TTP: time to progression; AE: adverse events; QoL: quality of life; BM: blood marker; IC: immune cell; SAS: Self-Rating Anxiety Scale; SDS: Self-Rating Depression Scale; QLQC30: Quality Of Life Questionnaire Core 30; CF: cardiac function; ECG: electrocardiogram; CK: creatinine kinase; CEF: cytoxan, epirubicin, and 5-fluorouracil; CAF: cytoxan, adriamycin, and 5-fluorouracil; TC-P: theprubicin, cytoxan, and paclitaxel; TAC: theprubicin, adriamycin, and cytoxan; AC-T: adriamycin, cytoxan, and theprubicin; CTF: cytoxan, theprubicin, and 5-fluorouracil; NT: navelbine and theprubicin. ^*∗*^Data were expressed as medium and interquartile range (IQR). #Details not reported.

**Table 2:** Secondary outcomes.

Outcomes	No. of trials	Heterogeneity		Effect size with 95% CI	Z with *P* value
		Chi-squared	*I*-squared (%)		
Immune cells					
CD3%	7^12,13,18,21,26−28^	514.87 (*P* < 0.00001)	99	3.71 (−3.85∼11.27)	0.96 (*P*=0.34)
CD4%	7^12,13,18,21,26−28^	1302.78 (*P* < 0.0001)	100	6.67 (−2.71∼16.06)	1.39 (*P*=0.16)
CD8%	7^12,13,18,21,26−28^	747.57 (*P* < 0.00001)	99	−0.97 (−7.54∼5.6)	0.29 (*P*=0.77)
CD4/CD8	7^12,13,18,21,26−28^	48.88 (*P* < 0.00001)	88	0.32 (0.07∼0.58)	2.5 (*P*=0.01
NK%	4^12,13,18,26^	29.39 (*P* < 0.00001)	90	5.54 (4.60∼6.47)	11.64 (*P* < 0.001)

Tumor markers					
CEA	1^13^	—	—	−2.39 (−3.99∼−0.79)	2.93 (*P*=0.003)
CA153	1^13^	—	—	−3.06 (−5.17∼−0.95)	2.85 (*P*=0.004)

**Table 3:** Safety of ADI.

Outcomes	No. of trials	Heterogeneity		Effect size with 95% CI	Z with *P* value
		Chi-squared	*I*-squared (%)		
Hepatic function					
AST	3^22,23,25^	112.69 (*P* < 0.00001)	98	−31.21 (−47.06∼−15.36)	3.86 (*P*=0.001)
ALT	3^22,23,25^	4.86 (*P*=0.09)	59	−4.04 (−5.57∼−2.51)	5.16 (*P* < 0.0001)
*γ*-GT	2^23,25^	0.08 (*P*=0.78)	0.0	−24.59 (−27.78∼−21.40)	15.1 (*P* < 0.0001)

Cardiac function					
CK-MB	2^22,23^	0.64 (*P*=0.42)	0.0	−4.04 (−5.91∼−2.17)	4.23 (*P* < 0.0001)
